# Cardiovascular disease risk and pathophysiology in South Asians: can longitudinal multi-omics shed light?

**DOI:** 10.12688/wellcomeopenres.16336.1

**Published:** 2020-10-27

**Authors:** Yan V. Sun, Chang Liu, Lisa Staimez, Mohammed K. Ali, Howard Chang, Dimple Kondal, Shivani Patel, Dean Jones, Viswanathan Mohan, Nikhil Tandon, Dorairaj Prabhakaran, Arshed A. Quyyumi, K. M. Venkat Narayan, Anurag Agrawal

**Affiliations:** 1 Department of Epidemiology, Rollins School of Public Health, Emory University, Atlanta, GA, 30322, USA; 2 Department of Biomedical Informatics, School of Medicine, Emory University, Atlanta, GA, 30322, USA; 3 Hubert Department of Global Health, Rollins School of Public Health, Emory University, Atlanta, GA, 30322, USA; 4 Department of Family and Preventive Medicine, School of Medicine, Emory University, Atlanta, GA, 30322, USA; 5 Department of Biostatistics and Bioinformatics, Rollins School of Public Health, Emory University, Atlanta, GA, 30322, USA; 6 Public Health Foundation of India, New Delhi, India; 7 Division of Pulmonary, Allergy, Critical Care and Sleep Medicine, Department of Medicine, School of Medicine, Emory University, Atlanta, GA, 30322, USA; 8 Madras Diabetes Research Foundation, Chennai, India; 9 All India Institute of Medical Sciences, New Delhi, India; 10 Department of Medicine, Division of Cardiology, Emory University School of Medicine, Atlanta, GA, 30322, USA; 11 Institute of Genomics and Integrative Biology, Council of Scientific and Industrial Research, New Delhi, India

**Keywords:** multi-omics, heart failure, atherosclerosis, subclinical CVD, HFpEF, HFrEF, South Asians, diabetes

## Abstract

Cardiovascular disease (CVD) is the leading cause of mortality in South Asia, with rapidly increasing prevalence of hypertension, type 2 diabetes and hyperlipidemia over the last two decades. Atherosclerotic CVD (ASCVD) affects South Asians earlier in life and at lower body weights, which is not fully explained by differential burden of conventional risk factors. Heart failure (HF) is a complex clinical syndrome of heterogeneous structural phenotypes including two major clinical subtypes, HF with preserved (HFpEF) and reduced ejection fraction (HFrEF). The prevalence of HF in South Asians is also rising with other metabolic diseases, and HFpEF develops at younger age and leaner body mass index in South Asians than in Whites. Recent genome-wide association studies, epigenome-wide association studies and metabolomic studies of ASCVD and HF have identified genes, metabolites and pathways associated with CVD traits. However, these findings were mostly driven by samples of European ancestry, which may not accurately represent the CVD risk at the molecular level, and the unique risk profile of CVD in South Asians. Such bias, while formulating hypothesis-driven research studies, risks missing important causal or predictive factors unique to South Asians. Importantly, a longitudinal design of multi-omic markers can capture the life-course risk and natural history related to CVD, and partially disentangle putative causal relationship between risk factors, multi-omic markers and subclinical and clinical ASCVD and HF. In conclusion, combining high-resolution untargeted metabolomics with epigenomics of rigorous, longitudinal design will provide comprehensive unbiased molecular characterization of subclinical and clinical CVD among South Asians. A thorough understanding of CVD-associated metabolomic profiles, together with advances in epigenomics and genomics, will lead to more accurate estimates of CVD progression and stimulate new strategies for improving cardiovascular health.

## Introduction

Cardiovascular disease (CVD) remains the most important cause of mortality worldwide. CVD is now the leading cause of mortality in India, accounting for 25% of deaths ^1, 2^. The prevalence of CVD risk factors including hypertension, type 2 diabetes, and lipid levels have increased rapidly over the last two decades ^3– 7^. Atherosclerotic CVD (ASCVD) phenotypes are heterogeneous ^8– 10^. The presence of subclinical ASCVD precursors like vascular dysfunction can be measured as endothelial dysfunction, arterial stiffness, or microvascular dysfunction. Subclinical structural changes can be detected by carotid intima-media thickening (CIMT), plaque deposition, coronary artery calcium (CAC), and reduced ankle-brachial index (ABI), all of which can predict future ASCVD events ^11– 18^. ASCVD affects South Asians earlier in life, with 52% of CVD deaths in individuals <70 years compared to 23% in the West, disparities that are not fully explained by differences in conventional risk factor burden ^19– 27^.

Heart failure (HF) is a complex clinical syndrome that results from structural and functional impairment of ventricular filling or output, and manifests itself in heterogeneous structural phenotypes (HF with preserved [HFpEF] or reduced ejection fraction [HFrEF]). HF prevalence in the US is projected to increase 46% from 2012 to 2030, resulting in over 8 million adults (≥18 years) with HF ^28^. The prevalence of HF in South Asians is also rising with other metabolic diseases ^29, 30^, and HFpEF develops at younger age and leaner BMI than for Whites ^31^.

## Genetic basis of atherosclerotic cardiovascular disease and heart failure

Genome-wide association studies (GWAS) have identified a large number of genetic loci associated with ASCVD ^32– 34^, HF ^35, 36^, and their risk factors ^37– 39^; however, these genetic variations explain only a small portion of risk in populations ^40, 41^. Joint genetic-environmental effects may be a key mechanism responsible for unexplained CVD risk. For example, environmental exposures can modify the gene expression levels through epigenetic mechanisms and this epigenetic modification can be inherited across cell generations to exert a long-term impact on the development of CVD.

In addition, current findings in CVD genetics are overwhelmingly driven by European ancestry, that disproportionally represents the majority of the global population at risk ^42^. Recent studies start showing the benefits of including diverse ancestries in discovering novel genetic loci associated with diseases ^43^, and improving the variance explained across ethnic groups. The South Asian population, who constitute over 20% of humanity, remain underrepresented in large genomic, epigenomic and other -omic studies. More GWAS in South Asians would improve both ethnicity-specific, and trans-ethnic discoveries of ASCVD- and HF-related loci, thus, address the high burden of these diseases in this large and growing population.

## Epigenetic signatures identify molecular pathways of CVD that are activated in the context of environmental risk

Epigenetics refers to molecular modifications that are unrelated to the primary DNA sequence and that can arise from environmental exposures ^44^. Epigenetic modification, through DNA methylation (DNAm) and other molecular mechanisms can regulate gene expression levels that can influence susceptibility to disease development ^45^, including atherosclerosis ^46^ and chronic inflammation ^47^, two important pathophysiological processes leading to CVD. Furthermore, epigenetic markers are modified by age ^48, 49^ and environmental risk factors, such as smoking ^50^ and poor nutrition ^51^. Thus, the epigenetic profile can capture many of the cumulative environmental effects that influence susceptibility to CVD and its adverse outcomes. An epigenome-wide association study (EWAS) is a study of epigenetic markers across the entire genome in a population exhibiting a specific trait ^52^. Thus, EWAS provides an unbiased approach for identifying molecular mediators of genetic and environmental factors that may explain residual risk of disease ^52^ and has been applied to investigate CVD and risk factors ^53– 57^. A very small number of EWAS have examined DNAm patterns associated with air pollution ^58, 59^, and to our knowledge, none has been reported from the South Asian setting despite being a very likely risk.

A recent EWAS of 11,461 individuals of European or African ancestry identified 52 DNAm sites significantly associated with incident coronary artery disease (CAD; n=1,895) using peripheral blood samples ^53^. This robust study reported epigenetic associations with effect sizes of a clinically relevant magnitude. Another EWAS identified 59 DNAm sites that associated with risk of dilated cardiomyopathy in left ventricular and peripheral blood samples ^57^. Bioinformatic analyses of differentially methylated regions showed enrichment for modification around binding sites for transcription factors involved in cardiac phenotypes. Studies in large populations have also identified DNAm sites associated with traditional CVD risk factors including age ^60– 62^, BMI ^63, 64^, diabetes ^65– 68^, blood lipids ^69^, smoking ^50, 70, 71^ and inflammation ^72^. These DNAm markers may provide a measure of longitudinal CVD risk ^73^, independently predicting future CVD events. However, DNAm sites need to be rigorously examined across genetically distinct cohorts. Since epigenomic profile can be modified due to changes in environmental and socio-behavioral factors, a longitudinal study can reveal the dynamics of the epigenomic profile, and potential causal or mediation effects in relation of CVD risk and progression. In sum, epigenetic profiles provide a signature of the cumulative burden of life-long exposure to CVD risks.

## Metabolomic signatures present complex metabolic state and environmental exposure

The metabolome is a global identification of all small molecules produced by cells during metabolism or obtained from environmental exposures. The metabolome thus provides a direct functional readout of cellular activity and physiologic status that can potentially be used for early disease identification, study of treatment effects, and for prognostication of disease progression ^74– 76^. It reflects the combined systemic effects of genetic, lifestyle, and environmental factors. Metabolomics is an emerging discipline that has the potential to transform the study of biological responses to environment exposures that underlie disease development. The untargeted metabolomic approach provides unbiased coverage of metabolites with greater breadth than targeted methods. Advances in untargeted metabolomics have increased the number of metabolites analyzed, thereby improving accuracy of disease detection and quantification ^77^. Metabolomics holds promise in elucidating interactions between genes and environment (such as air pollution) to uncover the pathophysiology and underlying molecular pathways of complex disorders such as ASCVD and HF.

Metabolomic research in humans has shown that modification or dysregulation of numerous metabolites, including amino acids, phospholipids, short-chain acylcarnitines, and nitric oxide synthesis, are associated with CVD risk and outcomes. In a study of 2,232 African and 1,366 European Americans from the Atherosclerosis Risk in Communities (ARIC) study (633 incident CAD cases), 19 metabolites collectively improved CAD risk prediction ^78^. Metabolomics study of dietary patterns among Asian Indians were found associated with cardiometabolic risk. A study of 145 Asian Indians in the Metabolic Syndrome and Atherosclerosis in South Asians Living in America (MASALA) pilot study revealed that the metabolite pattern of branched-chain amino acids, aromatic amino acids, and short-chain acylcarnitines, which are representative of a “Western/nonvegetarian” dietary pattern associated with adverse cardiometabolic profile ^79^. Recent studies also reported changes in global metabolism in relation to HF risk ^80^ and outcomes ^74^. A study of 515 HF patients identified a panel of metabolites that improved prediction of HF-related mortality and re-hospitalization, with variation between HF subgroups ^74, 81^. By demonstrating diagnostic and prognostic value in HF risk, these studies suggest that metabolomic research will distinguish HF subtypes.

## The multi-omics approach is critical to understand CVD risk at the molecular level

Genetics is one of the primary sources of epigenetic and metabolic variation ^52, 82, 83^. Recent GWAS have identified >160 genomic loci for CAD ^33^, 11 loci for HF ^36^, and >300 loci for type 2 diabetes ^84^. However, biological functions of most identified genetic loci remain unknown. Therefore, assessment of the functional linkage between identified epigenetic makers, metabolites, and genetic variants would be fruitful. Genomic data will complement epigenomic and metabolomic markers by identifying complex biological processes at the systems levels ^85^. Additionally, our recent joint epigenomics-metabolomics study of smoking ^86^ demonstrated that these multi-omic layers capture complementary components of biological systems in response to widespread risk exposures such as air pollution. By jointly analyzing genomic, epigenomic, and metabolomic profiles, we hypothesize that it could lead to identification of key genes and pathways that may be the molecular mediators of CVD. The molecular functions of identified epigenetic and metabolic markers, key genes and pathways involved in subclinical and clinical CVD will help us develop future targeted studies to improve comorbid disease prevention and clinical care strategies.

To extend this understanding to high-risk populations such as South Asians, it is important that such studies be performed in longitudinal cohorts that adequately represent the ethnicity and the environment. A longitudinal design enables the detection of changes in the characteristics of the target population at both the group and the individual level. Since the multi-omics profile (e.g., epigenome and metabolome) can be modified by dynamic and cumulative environmental factors, capturing the longitudinal multi-omics would benefit the understanding of risk development and progression of subclinical and clinical CVD in South Asians. Longitudinal changes of individual omic profile related to disease and aging have been documented in humans, primates and mice ^87– 89^. Longitudinal design can also distinguish cause from consequence using appropriate modeling ^90^. With large sample size, properly implemented mediation analysis and strong genetic instrumental variables for Mendelian Randomization analysis, future longitudinal studies in South Asian populations can further control for genetic and environmental confounders to better address causal relationship between -omic markers, environmental factors and CVD.

## Conclusion

Previous studies had limited coverage of metabolomics, had not addressed HFpEF heterogeneity, and none studied longitudinal profiles of ASCVD/HF or profiles in South Asians. Furthermore, no studies have employed the integrative multi-omic approach to disentangling complex molecular systems underlying ASCVD and HF. A thorough understanding of CVD-associated metabolomic profiles, together with advances in epigenomics and genomics, will lead to more accurate estimates of CVD progression and stimulate new strategies for improving cardiovascular health. Combining high-resolution untargeted metabolomics, epigenomics with a rigorous, longitudinal design would provide comprehensive molecular characterization of subclinical and clinical CVD among South Asians – a large global population with unique CVD patterns, and with potential variations in phenotypes. To fully understand the gene-environment interplay, it would also be useful to have studies of South Asians living in South Asia as well as South Asian emigrants.

## Data availability

No data are associated with this article.
