# Cardiovascular disease risk and pathophysiology in South Asians: can longitudinal multi-omics shed light?

**DOI:** 10.12688/wellcomeopenres.16336.2

**Published:** 2021-05-20

**Authors:** Yan V. Sun, Chang Liu, Lisa Staimez, Mohammed K. Ali, Howard Chang, Dimple Kondal, Shivani Patel, Dean Jones, Viswanathan Mohan, Nikhil Tandon, Dorairaj Prabhakaran, Arshed A. Quyyumi, K. M. Venkat Narayan, Anurag Agrawal

**Affiliations:** 1 Department of Epidemiology, Rollins School of Public Health, Emory University, Atlanta, GA, 30322, USA; 2 Department of Biomedical Informatics, School of Medicine, Emory University, Atlanta, GA, 30322, USA; 3 Hubert Department of Global Health, Rollins School of Public Health, Emory University, Atlanta, GA, 30322, USA; 4 Department of Family and Preventive Medicine, School of Medicine, Emory University, Atlanta, GA, 30322, USA; 5 Department of Biostatistics and Bioinformatics, Rollins School of Public Health, Emory University, Atlanta, GA, 30322, USA; 6 Public Health Foundation of India, New Delhi, India; 7 Division of Pulmonary, Allergy, Critical Care and Sleep Medicine, Department of Medicine, School of Medicine, Emory University, Atlanta, GA, 30322, USA; 8 Madras Diabetes Research Foundation, Chennai, India; 9 All India Institute of Medical Sciences, New Delhi, India; 10 Department of Medicine, Division of Cardiology, Emory University School of Medicine, Atlanta, GA, 30322, USA; 11 Institute of Genomics and Integrative Biology, Council of Scientific and Industrial Research, New Delhi, India

**Keywords:** multi-omics, heart failure, atherosclerosis, subclinical CVD, HFpEF, HFrEF, South Asians, diabetes

## Abstract

Cardiovascular disease (CVD) is the leading cause of mortality in South Asia, with rapidly increasing prevalence of hypertension, type 2 diabetes (T2DM) and hyperlipidemia over the last two decades. Atherosclerotic CVD (ASCVD) affects South Asians earlier in life and at lower body weights, which is not fully explained by differential burden of conventional risk factors. Heart failure (HF) is a complex clinical syndrome of heterogeneous structural phenotypes including two major clinical subtypes, HF with preserved (HFpEF) and reduced ejection fraction (HFrEF). The prevalence of HF in South Asians is also rising with other metabolic diseases, and HFpEF develops at younger age and leaner body mass index in South Asians than in Whites. Recent genome-wide association studies, epigenome-wide association studies and metabolomic studies of ASCVD and HF have identified genes, metabolites and pathways associated with CVD traits. However, these findings were mostly driven by samples of European ancestry, which may not accurately represent the CVD risk at the molecular level, and the unique risk profile of CVD in South Asians. Such bias, while formulating hypothesis-driven research studies, risks missing important causal or predictive factors unique to South Asians. Importantly, a longitudinal design of multi-omic markers can capture the life-course risk and natural history related to CVD, and partially disentangle putative causal relationship between risk factors, multi-omic markers and subclinical and clinical ASCVD and HF. In conclusion, combining high-resolution untargeted metabolomics with epigenomics of rigorous, longitudinal design will provide comprehensive unbiased molecular characterization of subclinical and clinical CVD among South Asians. A thorough understanding of CVD-associated metabolomic profiles, together with advances in epigenomics and genomics, will lead to more accurate estimates of CVD progression and stimulate new strategies for improving cardiovascular health.

## Introduction

Cardiovascular disease (CVD) remains the most important cause of mortality worldwide. CVD is now the leading cause of mortality in India, accounting for 25% of deaths ^1, 2^. The prevalence of CVD risk factors including hypertension, type 2 diabetes (T2DM), and lipid levels have increased rapidly over the last two decades ^3– 7^. Atherosclerotic CVD (ASCVD) phenotypes are heterogeneous ^8– 10^. The presence of subclinical ASCVD precursors like vascular dysfunction can be measured as endothelial dysfunction, arterial stiffness, or microvascular dysfunction. Subclinical structural changes can be detected by carotid intima-media thickening (CIMT), plaque deposition, coronary artery calcium (CAC), and reduced ankle-brachial index (ABI), all of which can predict future ASCVD events ^11– 18^. ASCVD affects South Asians earlier in life, with 52% of CVD deaths in individuals <70 years compared to 23% in the West, disparities that are not fully explained by differences in conventional risk factor burden ^19– 27^.

Heart failure (HF) is a complex clinical syndrome that results from structural and functional impairment of ventricular filling or output, and manifests itself in heterogeneous structural phenotypes (HF with preserved [HFpEF] or reduced ejection fraction [HFrEF]). HF prevalence in the US is projected to increase 46% from 2012 to 2030, resulting in over 8 million adults (≥18 years) with HF ^28^. The prevalence of HF in South Asians is also rising with other metabolic diseases ^29, 30^, and HFpEF develops at younger age and leaner BMI than for Whites ^31^.

Nearly half of the HF patients have HFpEF, with >90% being >60 years old, with rapidly increasing numbers ^32– 35^. Although numerous risk factors for HFpEF have been identified including hypertension, older age, female sex, obesity, diabetes, and renal disease ^36, 37^, there are currently no class I guideline recommended treatments for HFpEF that improve mortality ^38, 39^. HFpEF has received increased attention since HFpEF patients frequently experience delayed diagnosis and have limited treatment options. Recent studies have shown that HFpEF is a clinically heterogeneous disorder, consisting of subgroups with related comorbidities and pathophysiologies, which lead to different progression trajectories ^40, 41^. Employing latent class analytics and clustering techniques based on widely available clinical variables, several investigators have shown that patients with HFpEF can be divided into distinct classes with differing outcomes ^40, 41^.

CVD progression can be affected by both gene and environment via different molecular pathways and mechanisms. Although the high throughput technologies have enabled accurate and cost-effective genotyping in large population samples, comparable high throughput measurements of environmental exposures are needed for large population studies. Gene-environment interaction is a common mechanism to explain complex disease risk, and inter-individual variability. Better understanding of gene-environment interactions and their causal relationships will point to pathways and mechanisms as potential targets for treatment and intervention. The gene-environment interaction study may also help understand the CVD risk among immigrant populations exposure to different environmental and lifestyle factors.

In this paper, we focus on the current progress of omics studies on ASCVD and HF, as well as the anticipation of implications among South Asian population. A summary of selected CVD omics studies listed are shown in Table 1.

**Table 1.** Summary of selected omics studies of CVD.

PMID	First Author	Year	Phenotype	Sample size	Ethnicity
Genetic basis of atherosclerotic cardiovascular disease and heart failure					
33532862	Hartiala JA	2021	Myocardial infarction	Discovery ~61,000 cases, 577,000 controls Replication: ~165,000+27,000 subjects	European, East Asian
33020668	Koyama S	2020	CAD	121,234 cases, 527,824 controls	East Asian, European
N/A DOI:10.21203/ rs.3.rs-275591/v1	Assimes T	2021	CAD	~250,000 cases	European, African American, Hispanic, East Asian
26343387	Nikpay M	2015	CAD	60,801 cases, 123,504 controls	European, South Asian, East Asian, Hispanic, African American
29212778	van der Harst P	2018	CAD	Discovery: 34,541 cases, 261,984 controls Replication: 88,192 cases, 162,544 controls	European
31160810	Malik R	2018	Stroke	67,162 cases, 454,450 controls	European, East Asian, African, South Asian, mixed Asian, Latin American
31919418	Shah S	2020	HF	47,309 cases, 930,014 controls	European
23202125	Consortium CAD	2013	CAD	63,746 cases, 130,681 controls	European, South Asian
Epigenetic signatures identify molecular pathways of CVD that are activated in the context of environmental risk					
31424985	Agha G	2019	Myocardial infarction, CAD	11,461 subjects	European, African American
28515798	Nakatochi M	2017	Myocardial infarction	192 cases, 192 controls	Japanese
28172975	Rask-Andersen M	2016	Myocardial infarction	729 subjects	European
31615550	Westerman K	2019	CVD	Discovery: 2,023 subjects Validation: 2,587 subjects	European, African American, Hispanic
28838933	Meder B	2017	HF	Discovery: 41 cases, 31+31 controls Replication 1: 18+9 cases, 8+28 controls Replication 2: 82 cases, 109 controls	European
Proteomics and gene expression reveal the interactions between cell processes and external environment					
30587458	Bom MJ	2019	Coronary plaque morphology	196 subjects	European
32808014	Hoogeveen RM	2020	CVD	Discovery: 822 subjects Validation: 702 subjects	European
Metabolomic signatures present complex metabolic state and environmental exposure					
25881932	Cheng ML	2015	HF	Discovery: 183 cases, 51 controls Validation: 218 cases, 63 controls	East Asian
31092011	Wang Z	2019	CAD	3,598 subjects	African American, European
29893901	Bhupathiraju SN	2018	Cardiometabolic risk	145	South Asian
23788672	Zheng Y	2013	HF	1,744 subjects	African American
29096792	Lanfear DE	2017	HF	Discovery: 516 subjects Validation: 516 subjects	European, African American
The multi-omics approach is critical to understand CVD risk at the molecular level					
29096792	Andersson C	2019	HF	8,372 subjects	European
33836805	Palou-Marquez G	2021	CVD	Methylation: 2,055 subjects Gene expression: 914 subjects	European

## Genetic basis of atherosclerotic cardiovascular disease and heart failure

Genome-wide association studies (GWAS) have identified a large number of genetic loci associated with coronary heart disease ^42– 44^, ASCVD ^45- 47^, HF ^48, 49^, and their risk factors, such as BMI ^50, 51^, blood lipids ^52, 53^, blood pressure ^54– 57^, and T2DM ^58^; however, these genetic variations explain only a small portion of risk in populations ^59, 60^. Joint genetic-environmental effects may be a key mechanism responsible for unexplained CVD risk. For example, environmental exposures can modify the gene expression levels through epigenetic mechanisms and this epigenetic modification can be inherited across cell generations to exert a long-term impact on the development of CVD.

A large number of genetic associations have been identified in large population studies for CVD and risk factors. Although identified genetic associations have small effect sizes individually, polygenetic risk score (PRS) can combine such individual effects into a much stronger predicter of a disease trait. A few studies have shown the successful identifications of CAD and relevant traits ^61, 62^. Earlier studies of PRS showed that the European ancestry-based can be transferred in other ancestry groups including South Asians, the associations of European ancestry-derived PRS were typically weaker in non-European ancestries. With large GWAS results from multiple ancestry groups, the PRS can be optimized to present ancestry-specific genetic risk for CVD. A study of 7,244 South Asian UK Biobank participants derived a PRS of CAD for South Asians from the previous GWAS findings that are primarily European-based. The PRS included 6,630,150 common variants, and demonstrated a successful framework for developing ancestry-specific PRS ^63^. In another study, researchers identified significant association between the GRS, which comprised of 29 genome-wide significant blood pressure variants found among European descent, and blood pressure among South Asians ^54^.

Current findings in CVD genetics are overwhelmingly driven by European ancestry, that disproportionally represents the majority of the global population at risk ^64^. Recent studies start showing the benefits of including diverse ancestries in discovering novel genetic loci associated with diseases ^65^, and improving the variance explained across ethnic groups. The South Asian population, who constitute over 20% of humanity, remain underrepresented in large genomic, epigenomic and other -omic studies. More GWAS in South Asians would improve both ethnicity-specific, and trans-ethnic discoveries of ASCVD- and HF-related loci, thus, address the high burden of these diseases in this large and growing population.

## Epigenetic signatures identify molecular pathways of CVD that are activated in the context of environmental risk

Epigenetics refers to molecular modifications that are unrelated to the primary DNA sequence and that can arise from environmental exposures ^66^. Epigenetic modification, through DNA methylation (DNAm) and other molecular mechanisms can regulate gene expression levels that can influence susceptibility to disease development ^67^, including atherosclerosis ^68^ and chronic inflammation ^69^, two important pathophysiological processes leading to CVD. Furthermore, epigenetic markers are modified by age ^70, 71^ and environmental risk factors, such as smoking ^72^ and poor nutrition ^73^. Thus, the epigenetic profile can capture many of the cumulative environmental effects that influence susceptibility to CVD and its adverse outcomes. An epigenome-wide association study (EWAS) is a study of epigenetic markers across the entire genome in a population exhibiting a specific trait ^74^. EWAS provides an unbiased approach for identifying molecular mediators of genetic and environmental factors that may explain residual risk of disease ^74^ and has been applied to investigate CVD ^75– 79^ and risk factors ^80– 85^.

A recent EWAS of 11,461 individuals of European or African ancestry identified 52 DNAm sites significantly associated with incident coronary artery disease (CAD; n=1,895) using peripheral blood samples ^75^. This robust study reported epigenetic associations with effect sizes of a clinically relevant magnitude. Another EWAS identified 59 DNAm sites that associated with risk of dilated cardiomyopathy in left ventricular and peripheral blood samples ^79^. Bioinformatic analyses of differentially methylated regions showed enrichment for modification around binding sites for transcription factors involved in cardiac phenotypes. Studies in large populations have also identified DNAm sites associated with traditional CVD risk factors including age ^70, 86, 87^, BMI ^88, 89^, diabetes ^80, 81, 90, 91^, blood lipids ^92^, smoking ^72, 93, 94^ and inflammation ^95^. These DNAm markers may provide a measure of longitudinal CVD risk ^96^, independently predicting future CVD events. However, DNAm sites need to be rigorously examined across genetically distinct cohorts. The study of incident T2DM conducted among Indian Asians and Europeans showed a 2.5 times higher adjusted risk among Indian Asians than Europeans, and five loci including *ABCG1*, *PHOSPHO1*, *SOCS3*, *SREBF1*, and *TXNIP* were associated with incident T2DM among Indian Asians and replicated among Europeans ^81^. The other study of incident T2DM reported additional loci such as *PHGDH* and *CPT1A*, which were discovered among Europeans and replicated among Indian Asians ^80^. The study of blood pressure compared the methylation profiles between Europeans and South Asians revealed many distinct loci between the two ancestries with a small overlap ^84^. To our current knowledge, some known trans-ethic epigenetic loci might be transportable from Europeans to South Asians, but there is still a lack in studies specifically among large South Asian populations to further explore novel loci to explain the higher risk. A number of EWAS have examined DNAm patterns associated with air pollution, which is a known environmental risk factor for CVD ^97, 98^. Since epigenomic profile can be modified due to changes in environmental and socio-behavioral factors, a longitudinal study can reveal the dynamics of the epigenomic profile, and potential causal or mediation effects in relation of CVD risk and progression. In sum, epigenetic profiles provide a signature of the cumulative burden of life-long exposure to CVD risks.

## Proteomics and gene expression reveal the interactions between cell processes and external environment

The proteomics technologies have evolved rapidly in the past two decades. Recent applications of proteomics in population studies have produced interesting findings in CVD research ^99^. A recent study of the healthy human heart tissue collected from autopsy determined the healthy heart proteome. The resulted database, which included over 10,700 proteins, is a comprehensive resource for the downstream investigations ^100^. In a targeted proteomics study, two protein signatures for high-risk plaques and absence of coronary atherosclerosis were identified among a cohort with suspected coronary artery disease ^101^. The prediction accuracy of a model constructed by 50 proteins showed a better prediction accuracy in adverse cardiovascular events than a traditional risk factor model ^102^. These findings are critical in CVD risk prediction and differentiation of CVD subtypes. In addition, gene expression profiling in CVD also enables a better understanding of pathophysiology of CVD ^103^. Patterns of gene expression of human aorta tissue was investigated to identify genes with prediction power in atherosclerosis ^104^. Studies of non-coding transcriptome, which even though have limited protein-coding functions, are discovering the pathology of CVD. To eventually realize precision medicine, we are still facing the challenges of standardization of methodologies and translation ^105^. In addition, researches in this area largely depend on the availability of tissues from autopsy, thus the clinical translation and implementation has been limited. However, such studies facilitate a better understanding on the disease causal pathway and mechanism. Particularly, there has been a lack of similar researches particularly among South Asians, which is urging the future efforts of investigation.

## Metabolomic signatures present complex metabolic state and environmental exposure

The metabolome is a global identification of all small molecules produced by cells during metabolism or obtained from environmental exposures. The metabolome thus provides a direct functional readout of cellular activity and physiologic status that can potentially be used for early disease identification, study of treatment effects, and for prognostication of disease progression ^106– 108^. It reflects the combined systemic effects of genetic, lifestyle, and environmental factors. Metabolomics is an emerging discipline that has the potential to transform the study of biological responses to environment exposures that underlie disease development. The untargeted metabolomic approach provides unbiased coverage of metabolites with greater breadth than targeted methods. Advances in untargeted metabolomics have increased the number of metabolites analyzed, thereby improving accuracy of disease detection and quantification ^109^. Metabolomics holds promise in elucidating interactions between genes and environment (such as air pollution) to uncover the pathophysiology and underlying molecular pathways of complex disorders such as ASCVD and HF.

Metabolomic research in humans has shown that modification or dysregulation of numerous metabolites, including amino acids, phospholipids, short-chain acylcarnitines, and nitric oxide synthesis, are associated with CVD risk and outcomes. In a study of 2,232 African and 1,366 European Americans from the Atherosclerosis Risk in Communities (ARIC) study (633 incident CAD cases), 19 metabolites collectively improved CAD risk prediction ^110^. Metabolomics study of dietary patterns among Asian Indians were found associated with cardiometabolic risk. A study of 145 Asian Indians in the Metabolic Syndrome and Atherosclerosis in South Asians Living in America (MASALA) pilot study revealed that the metabolite pattern of branched-chain amino acids, aromatic amino acids, and short-chain acylcarnitines, which are representative of a “Western/nonvegetarian” dietary pattern associated with adverse cardiometabolic profile ^111^. Recent studies also reported changes in global metabolism in relation to HF risk ^112^ and outcomes ^106^. A study of 515 HF patients identified a panel of metabolites that improved prediction of HF-related mortality and re-hospitalization, with variation between HF subgroups ^106, 113^. By demonstrating diagnostic and prognostic value in HF risk, these studies suggest that metabolomic research will distinguish HF subtypes.

## The multi-omics approach is critical to understand CVD risk at the molecular level

Genetics is one of the primary sources of epigenetic and metabolic variation ^74, 114, 115^. Recent GWAS have identified >160 genomic loci for CAD ^46^, 11 loci for HF ^49^, and >300 loci for T2DM ^58^. However, biological functions of most identified genetic loci remain unknown. Therefore, assessment of the functional linkage between identified epigenetic makers, metabolites, and genetic variants would be fruitful. Unlike the genome profile, epigenomic, transcriptomic, proteomic and metabolomic profiles can be modified by environmental exposures, physiological conditions and disease status. Genomic data will complement epigenomic and metabolomic markers by identifying complex biological processes at the systems levels ^116^. Current omics studies predominantly use a cross-sectional design, which enables the novel biomarker discovery but is limited to infer causal associations due to confounding and revers causation. A longitudinal design including baseline omics and incident CVD would better demonstrate the prediction utility of omics markers for CVD progression, and has been implemented in omic association studies in other ancestry groups. However, such a design doesn’t incorporate the longitudinal changes of omics markers in relation to varying environment, pathobiology and disease progression. Repeated measurements of omics profiles excluding genomics would be important to understand long-term risk and natural history of chronic diseases ^117^ such as CVD. Such omics changes may also help identify reversible targets for novel interventions for CVD outcomes.

A recent study using longitudinal big data including genome, immunome, transcriptome, proteome, metabolome, microbiome and wearable monitoring have showed the potential of revealing cardiovascular pathophysiology on a molecular basis ^118^. Although increasing number of population studies have measured multi-omics data, recent studies have focused on single -omic association study and used additional -omics data to better understand the molecular functions of identified associations ^119^. Integrated multi-omics studies of CVD outcomes have been limited. A large HF study of over 8,000 participants within the Framingham Heart Study utilized integrative trans-omics data including genetic variations, DNA methylation, and gene expression data to reveal genetic contributions towards HF. The transportability of such findings to South Asians needs to be evaluated ^120^. Another study integrating DNA methylation and gene expression data identified independent latent factors associated with CVD. The unsupervised machine learning of multi-omics successfully improved classification and discrimination ^121^. Additionally, our recent joint epigenomics-metabolomics study of smoking ^122^ demonstrated that these multi-omic layers capture complementary components of biological systems in response to widespread risk exposures such as air pollution. By jointly analyzing genomic, epigenomic, and metabolomic profiles, we hypothesize that it could lead to identification of key genes and pathways that may be the molecular mediators of CVD. The molecular functions of identified epigenetic and metabolic markers, key genes and pathways involved in subclinical and clinical CVD will help us develop future targeted studies to improve comorbid disease prevention and clinical care strategies.

To extend this understanding to high-risk populations such as South Asians, it is important that such studies be performed in longitudinal cohorts that adequately represent the ethnicity and the environment. A longitudinal design enables the detection of changes in the characteristics of the target population at both the group and the individual level. Since the multi-omics profile (e.g., epigenome and metabolome) can be modified by dynamic and cumulative environmental factors, capturing the longitudinal multi-omics would benefit the understanding of risk development and progression of subclinical and clinical CVD in South Asians. Longitudinal changes of individual omic profile related to disease and aging have been documented in humans, primates and mice ^123– 125^. Longitudinal design can also distinguish cause from consequence using appropriate modeling ^126^. With large sample size, properly implemented mediation analysis and strong genetic instrumental variables for Mendelian Randomization analysis, future longitudinal studies in South Asian populations can further control for genetic and environmental confounders to better address causal relationship between -omic markers, environmental factors and CVD.

## Strategies of omics studies

Due to the rapidly growing technologies such as next generation sequencing and untargeted metabolomics, massive amount of accurate data can be obtained in a cost-effective manner. Therefore, there has been an urgent need for new methodologies and algorithms to form, for the purposes of computationally intensive data managing and analysis ^116^. For GWAS, tools such as PLINK ^127^, RVTESTS ^128^ and GENESIS ^129^ now allow the incorporation of many data pieces such as genotypes, annotations, allele frequencies and phenotypes to be processed efficiently in particular formats. For untargeted MWAS, mixOmics ^130^ and xmsPANDA ( https://github.com/kuppal2/xmsPANDA) has been utilized to conduct feature selection, and the software Mummichog ^131^ for pathway exploration based on untargeted metabolomics data has been made available to bypass the feature identification stage. In addition, various tools have been developed for network analysis. For example, the weighted gene co-expression network analysis can be implemented using WGCNA ^132^ to identify clustering of genes, the software xMWAS ^133^ can perform the integration and differential network analysis for multiple layers of omics data. The evolving omics researches of cardiovascular risk are coupled with these tools to achieve better understating of the disease biological mechanism and pathway.

## The multi-omics approach may address several challenges in CVD research facing South Asians

South Asians are an understudied population at high CVD risk even at low body weight and young ages, with a high propensity to diabetes, dyslipidemia, and hepatic steatosis, features that may provide new insights into CVD risk in the presence and absence of traditional risk factors. Given the genetic and molecular underpinning of CVD and risk factors, the genetic and molecular markers identified from multi-omics research may help accurately profile the CVD risk, given the unique characteristics among South Asians. Secondly, the multi-omics approach also holds the promise of revealing the heterogeneous mechanisms of CVD and risk factors. For example, HFpEF patients can be clustered into distinct subclasses with different clinical outcome using available demographic and clinical variables ^40, 41^. T2DM is also a multi-factorial disease that involves numerous genetic pathways and many environmental factors ^134^, and has high prevalence in South Asian populations. Recent studies of T2MD subgroups have revealed striking heterogeneity of T2DM risk, etiology and outcomes, which may be further illustrated by multi-omic profiling. Lastly, multi-omics can help explain inter-individual variability in response to environmental risk exposures. Exposures to environmental risk such as air pollution (common and severer CVD risk in South Asia) are multifactorial and time-varying, thus, their measurements can be incomplete, costly, and imprecise for pathophysiological effects. Omic technologies such as epigenomics and metabolomics represent exogenous and endogenous effects related to CVD risk and have emerged as key components of exposome measures ^135, 136^. They can capture the biological response to environmental exposures, thus, providing more precise risk assessment for subclinical and clinical CVD. Improved omics measurements of environmental exposure will also enable large scale gene-environment interaction study to further uncover molecular mechanisms underlying CVD pathophysiology.

In addition to common genomic and multi-omics factors of CVD across ancestry groups, studying multi-omics among large population samples within South Asians may also discover genetic variants or molecular signatures unique for South Asians. Incorporating these multi-omic profiles can optimize the diagnosis, treatment and prognosis of CVD, which is the most important and still growing health burden for South Asian populations.

## Conclusion

Despite the available tools and fruitful research conducted among European ancestry, the implementation of similar research among South Asians remains challenging. Large high-quality studies are needed, with the requirements of high volume recruiting of population-based study samples, well-defined disease and phenotypes, long time follow-up, establishment of data registries, close collaborations between scientists with various expertise, such as study design, molecular biology and bioinformatics. There is a growing effort of research among the South Asian population, such as the Center for cArdio-metabolic Risk Reduction in South Asia (CARRS) study ^137^, which is a large longitudinal study of 28,000 subjects across three large cities in South Asia. Studies that employ the integrative multi-omic approach to disentangling complex molecular systems underlying CVD are still anticipated. A thorough understanding of CVD-associated metabolomic profiles, together with advances in epigenomics and genomics, will lead to more accurate estimates of CVD progression and stimulate new strategies for improving cardiovascular health. Combining high-resolution untargeted metabolomics, epigenomics with a rigorous, longitudinal design would provide comprehensive molecular characterization of subclinical and clinical CVD among South Asians – a large global population with unique CVD patterns, and with potential variations in phenotypes. To fully understand the gene-environment interplay, it would also be useful to have studies of South Asians living in South Asia as well as South Asian emigrants.

## Data availability

No data are associated with this article.
