# Supplementary material for: Cardiovascular disease risk and pathophysiology in South Asians: can longitudinal multi-omics shed light?
Source: Wellcome Open Res. 2021 May 20;5:255. Originally published 2020 Oct 27. [Version 2] doi: 10.12688/wellcomeopenres.16336.2 (PMC8176264; doi:10.12688/wellcomeopenres.16336.2)
Supplement: Peer review history

# Peer review: Reviewer response for version 2

**DOI:** 10.21956/wellcomeopenres.18643.r43985

**Published:** 2021-06-03

**Authors:** Chunyu Liu

**Affiliations:** 1 Department of Biostatistics, Boston University, Boston, MA, USA

I believe the authors have adequately addressed my comments. Thank you.

Is the review written in accessible language?

Yes

Are all factual statements correct and adequately supported by citations?

Yes

Are the conclusions drawn appropriate in the context of the current research literature?

Yes

Is the topic of the review discussed comprehensively in the context of the current literature?

Partly

Reviewer Expertise:

Statistical genetics and genomics.

I confirm that I have read this submission and believe that I have an appropriate level of expertise to confirm that it is of an acceptable scientific standard.

# Peer review: Reviewer response for version 2

**DOI:** 10.21956/wellcomeopenres.18643.r43986

**Published:** 2021-05-24

**Authors:** Nilay Shah

**Affiliations:** 1 Division of Cardiology/Department of Medicine and Department of Preventive Medicine, Northwestern University Feinberg School of Medicine, Chicago, IL, USA

The authors have satisfactorily revised the article in response to reviewer comments.

Is the review written in accessible language?

Yes

Are all factual statements correct and adequately supported by citations?

Yes

Are the conclusions drawn appropriate in the context of the current research literature?

Partly

Is the topic of the review discussed comprehensively in the context of the current literature?

No

Reviewer Expertise:

Cardiovascular disease epidemiology, South Asian cardiovascular health and disease prevention.

I confirm that I have read this submission and believe that I have an appropriate level of expertise to confirm that it is of an acceptable scientific standard.

# Peer review: Reviewer response for version 1

**DOI:** 10.21956/wellcomeopenres.17960.r41637

**Published:** 2020-12-09

**Authors:** Nilay Shah

**Affiliations:** 1 Division of Cardiology/Department of Medicine and Department of Preventive Medicine, Northwestern University Feinberg School of Medicine, Chicago, IL, USA

In the review article “Cardiovascular disease risk and pathophysiology in South Asians: can longitudinal multi-omics shed light?” by Sun et al., the authors provide a summary of research findings from genetic, epigenetic, and metabolomic studies of cardiovascular risk factors and cardiovascular disease. The narrative begins to detail how these “multi-omic” approaches may be used to better understand the mechanisms of cardiovascular diseases, and concludes with a recommendation to increase “multi-omics” research in the South Asian population, with particular emphasis on longitudinal epigenomics and metabolomics to comprehensively characterize subclinical and clinical cardiovascular disease at the molecular level.

 The authors have identified and described an important and growing field of research that is relevant to South Asians, a population that is disproportionately affected by cardiovascular diseases. A number of comments and suggests are submitted for the authors’ consideration to strengthen the relevance, clarity, and impact of their review:

  

1. The narrative review of “multi-omics” provided by the authors is superficial, and does not sufficiently detail these growing methodologies. The authors are encouraged to broaden their narrative of these -omics tools, including recent findings and how these tools are being used to develop strategies to improve cardiovascular health in European ancestry populations (which the authors acknowledge are the populations for which most data are available). This broader narrative and contextualization would more comprehensively describe the current state of this field, and help the reader understand how -omics approaches may be applied in South Asians.
  For instance, recent GWAS have been conducted in South Asian diaspora populations, and have contributed to a variety of strategies to understand cardiovascular risk in this population such as polygenic risk scoring (e.g., Wang M et al. “Validation of a genome-wide polygenic risk score for coronary artery disease in South Asians.” J Am Coll Cardiol. 2020;76(6):703-14 ^1^.) This and other recent similar studies would be helpful to include.
2. Since the narrative review is cursory, it is not clear how the authors envision “multi-omic” methodologies should be deployed in South Asian populations. What are the key research questions with these methods in the South Asian population? What do the authors hypothesize will be found? Are there any characteristics specific to the South Asian population that should be accounted for (for example, emerging epidemiologic work identifies South Asian subgroups, e.g. Pakistani, Indian, etc.; how does this fit into research evaluating environmental influences on epigenomics or metabolomics)? Addressing these questions seems important for this review to understand how longitudinal multi-omics might “shed light” on CVD in South Asians.
3. Are longitudinal -omics a component of research in other (including European ancestry) populations? What insights have been gained from such work? The authors suggest a longitudinal -omics approach in their concluding paragraphs, but have inadequately supported the potential merits of this strategy.
4. This review would be a prime opportunity to move this field forward beyond only stating that these -omics tools should be applied in South Asian populations. What are the existing challenges/barriers preventing these tools from being implemented in the study of South Asian CVD and CVD risk? (In parallel, why have most studies using -omics approaches occurred in European ancestry populations?) What strategies would help overcome these barriers? The authors are recommended to consider addressing these questions in their review to provide important context to the state of the science and a roadmap to achieve their recommended future directions.
5. The authors’ concluding paragraph introduces new concepts that were not previously discussed (for example, “HFpEF heterogeneity,” and the differentiation of South Asians in South Asia versus diaspora populations, which certainly would seem relevant with respect to environment-gene interaction). Consequently, their conclusions seem to be obscured and do not follow from their narrative. I recommended discussing these components of research gaps in South Asians earlier in the review, in order to justify their conclusions.

Is the review written in accessible language?

Yes

Are all factual statements correct and adequately supported by citations?

Yes

Are the conclusions drawn appropriate in the context of the current research literature?

Partly

Is the topic of the review discussed comprehensively in the context of the current literature?

No

Reviewer Expertise:

Cardiovascular disease epidemiology, South Asian cardiovascular health and disease prevention

I confirm that I have read this submission and believe that I have an appropriate level of expertise to confirm that it is of an acceptable scientific standard, however I have significant reservations, as outlined above.

## References

[1] : Validation of a Genome-Wide Polygenic Score for Coronary Artery Disease in South Asians. *J Am Coll Cardiol* .2020;76(6) : 10.1016/j.jacc.2020.06.024 703-714 10.1016/j.jacc.2020.06.024 32762905PMC7592606

# Response

**Published:** 2021-05-07

**Authors:** Yan Sun

**Affiliations:** Emory University Rollins School of Public Health, USA

*1. The narrative review of “multi-omics” provided by the authors is superficial, and does not sufficiently detail these growing methodologies. The authors are encouraged to broaden their narrative of these -omics tools, including recent findings and how these tools are being used to develop strategies to improve cardiovascular health in European ancestry populations (which the authors acknowledge are the populations for which most data are available). This broader narrative and contextualization would more comprehensively describe the current state of this field, and help the reader understand how -omics approaches may be applied in South Asians.*

*For instance, recent GWAS have been conducted in South Asian diaspora populations, and have contributed to a variety of strategies to understand cardiovascular risk in this population such as polygenic risk scoring (e.g., Wang M et al. “Validation of a genome-wide polygenic risk score for coronary artery disease in South Asians.” J Am Coll Cardiol. 2020;76(6):703-141.) This and other recent similar studies would be helpful to include.*

**We appreciate the comments and suggestions. In response to reviewer 1’s comments, we included a summary (Table 1) of recent omics studies of CVD in European ancestry and multi-ethnic populations. We also summarized growing methodologies and analytical tools which could be used to improve cardiovascular health among South Asians. We have added the following paragraph of “Strategies of omics studies” in the revised manuscript paper as below:**

 "Due to the rapidly growing technologies such as next generation sequencing and untargeted metabolomics, massive amount of accurate data can be obtained in a cost-effective manner. Therefore, there has been an urgent need for new methodologies and algorithms to form, for the purposes of computationally intensive data managing and analysis.[116] For GWAS, tools such as PLINK,[127] RVTESTS[128] and GENESIS[129] now allow the incorporation of many data pieces such as genotypes, annotations, allele frequencies and phenotypes to be processed efficiently in particular formats. For untargeted MWAS, mixOmics[130] and xmsPANDA (https://github.com/kuppal2/xmsPANDA) has been utilized to conduct feature selection, and the software Mummichog[131] for pathway exploration based on untargeted metabolomics data has been made available to bypass the feature identification stage. In addition, various tools have been developed for network analysis. For example, the weighted gene co-expression network analysis can be implemented using WGCNA[132] to identify clustering of genes, the software xMWAS[133] can perform the integration and differential network analysis for multiple layers of omics data. The evolving omics researches of cardiovascular risk are coupled with these tools to achieve better understating of the disease biological mechanism and pathway."

**We agree that the polygenic risk score study by Wang M et al is a great example showing how to use existing GWAS findings and data of CAD to optimize the utility in South Asians. We added the following discussion about polygenic risk score in the “Genetic basis of atherosclerotic cardiovascular disease and heart failure” section as below:**

 "A large number of genetic associations have been identified in large population studies for CVD and risk factors. Although identified genetic associations have small effect sizes individually, polygenetic risk score (PRS) can combine such individual effects into a much stronger predicter of a disease trait. A few studies have shown the successful identifications of CAD and relevant traits.[61, 62] Earlier studies of PRS showed that the European ancestry-based can be transferred in other ancestry groups including South Asians, the associations of European ancestry-derived PRS were typically weaker in non-European ancestries. With large GWAS results from multiple ancestry groups, the PRS can be optimized to present ancestry-specific genetic risk for CVD.  A study of 7,244 South Asian UK Biobank participants derived a PRS of CAD for South Asians from the previous GWAS findings that are primarily European-based. The PRS included 6,630,150 common variants, and demonstrated a successful framework for developing ancestry-specific PRS.[63] In another study, researchers identified significant association between the GRS, which comprised of 29 genome-wide significant blood pressure variants found among European descent, and blood pressure among South Asians.[54]"

*2. Since the narrative review is cursory, it is not clear how the authors envision “multi-omic” methodologies should be deployed in South Asian populations. What are the key research questions with these methods in the South Asian population? What do the authors hypothesize will be found? Are there any characteristics specific to the South Asian population that should be accounted for (for example, emerging epidemiologic work identifies South Asian subgroups, e.g. Pakistani, Indian, etc.; how does this fit into research evaluating environmental influences on epigenomics or metabolomics)? Addressing these questions seems important for this review to understand how longitudinal multi-omics might “shed light” on CVD in South Asians.*

**We appreciate reviewer’s suggestion. We included a new section of “The multi-omics approach may address several challenges in CVD research facing South Asians” to discuss three main research questions as initial examples for future multi-omics research in South Asians as below:**

 "South Asians are an understudied population at high CVD risk even at low body weight and young ages, with a high propensity to diabetes, dyslipidemia, and hepatic steatosis, features that may provide new insights into CVD risk in the presence and absence of traditional risk factors. Given the genetic and molecular underpinning of CVD and risk factors, the genetic and molecular markers identified from multi-omics research may help accurately profile the CVD risk, given the unique characteristics among South Asians. Secondly, the multi-omics approach also holds the promise of revealing the heterogeneous mechanisms of CVD and risk factors. For example, HFpEF patients can be clustered into distinct subclasses with different clinical outcome using available demographic and clinical variables.[40, 41] T2DM is also a multi-factorial disease that involves numerous genetic pathways and many environmental factors,[134] and has high prevalence in South Asian populations. Recent studies of T2MD subgroups have revealed striking heterogeneity of T2DM risk, etiology and outcomes, which may be further illustrated by multi-omic profiling. Lastly, multi-omics can help explain inter-individual variability in response to environmental risk exposures. Exposures to environmental risk such as air pollution (common and severer CVD risk in South Asia) are multifactorial and time-varying, thus, their measurements can be incomplete, costly, and imprecise for pathophysiological effects. Omic technologies such as epigenomics and metabolomics represent exogenous and endogenous effects related to CVD risk and have emerged as key components of exposome measures.[135, 136] They can capture the biological response to environmental exposures, thus, providing more precise risk assessment for subclinical and clinical CVD. Improved omics measurements of environmental exposure will also enable large scale gene-environment interaction study to further uncover molecular mechanisms underlying CVD pathophysiology." 

 "In addition to common genomic and multi-omics factors of CVD across ancestry groups, studying multi-omics among large population samples within South Asians may also discover genetic variants or molecular signatures unique for South Asians. Incorporating these multi-omic profiles can optimize the diagnosis, treatment and prognosis of CVD, which is the most important and still growing health burden for South Asian populations."

*3. Are longitudinal -omics a component of research in other (including European ancestry) populations? What insights have been gained from such work? The authors suggest a longitudinal -omics approach in their concluding paragraphs, but have inadequately supported the potential merits of this strategy.*

**Unlike the genome profile, epigenomic, transcriptomic, proteomic and metabolomic profiles can be modified by environmental exposures, physiological conditions and disease status. The need for longitudinal multi-omics includes two components. First, current omics studies predominantly use a cross-sectional design, which enables the novel biomarker discovery but is limited to infer causal associations due to confounding and reverse causation. A longitudinal design including baseline omics and incident CVD would better demonstrate the prediction utility of omics markers for CVD progression, and has been implemented in omic association studies in other ancestry groups. Secondly, repeated measurement of omics profiles excluding genomics would be important to understand long-term risk and natural history of chronic diseases (PMID 22424236) such as CVD, since these omics layers can capture the varying environment and pathobiology related to CVD development. Such omics changes may also help identify reversible targets for novel interventions for CVD outcomes. However, the multi-omics study with repeated measures hasn’t been reported for CVD. We added the following discussion to illustrate the need for longitudinal multi-omics in CVD research in the section of “The multi-omics approach is critical to understand CVD risk at the molecular level” as below:**

 "Unlike the genome profile, epigenomic, transcriptomic, proteomic and metabolomic profiles can be modified by environmental exposures, physiological conditions and disease status. Genomic data will complement epigenomic and metabolomic markers by identifying complex biological processes at the systems levels.[116] Current omics studies predominantly use a cross-sectional design, which enables the novel biomarker discovery but is limited to infer causal associations due to confounding and revers causation. A longitudinal design including baseline omics and incident CVD would better demonstrate the prediction utility of omics markers for CVD progression, and has been implemented in omic association studies in other ancestry groups. However, such a design doesn’t incorporate the longitudinal changes of omics markers in relation to varying environment, pathobiology and disease progression. Repeated measurements of omics profiles excluding genomics would be important to understand long-term risk and natural history of chronic diseases[117] such as CVD. Such omics changes may also help identify reversible targets for novel interventions for CVD outcomes."

*4. This review would be a prime opportunity to move this field forward beyond only stating that these -omics tools should be applied in South Asian populations. What are the existing challenges/barriers preventing these tools from being implemented in the study of South Asian CVD and CVD risk? (In parallel, why have most studies using -omics approaches occurred in European ancestry populations?) What strategies would help overcome these barriers? The authors are recommended to consider addressing these questions in their review to provide important context to the state of the science and a roadmap to achieve their recommended future directions.*

**We appreciate the suggestion. We revised the manuscript to address existing challenges and barriers for implementing multi-omics studies in South Asians in “Conclusion” section as below:**

 "Despite the available tools and fruitful research conducted among European ancestry, the implementation of similar research among South Asians remains challenging. Large high-quality studies are needed, with the requirements of high volume recruiting of population-based study samples, well-defined disease and phenotypes, long time follow-up, establishment of data registries, close collaborations between scientists with various expertise, such as study design, molecular biology and bioinformatics. There is a growing effort of research among the South Asian population, such as the Center for cArdio-metabolic Risk Reduction in South Asia (CARRS) study,[137] which is a large longitudinal study of 28,000 subjects across three large cities in South Asia. Studies that employ the integrative multi-omic approach to disentangling complex molecular systems underlying CVD are still anticipated."

*5. The authors’ concluding paragraph introduces new concepts that were not previously discussed (for example, “HFpEF heterogeneity,” and the differentiation of South Asians in South Asia versus diaspora populations, which certainly would seem relevant with respect to environment-gene interaction). Consequently, their conclusions seem to be obscured and do not follow from their narrative. I recommended discussing these components of research gaps in South Asians earlier in the review, in order to justify their conclusions.*

**We included background of HFpEF heterogeneity and the role of gene-environment interaction in the “Introduction” section as below. We also discussed the research gaps in a new section in response to comment #2.**

 "Nearly half of the HF patients have HFpEF, with >90% being >60 years old, with rapidly increasing numbers.[32-35] Although numerous risk factors for HFpEF have been identified including hypertension, older age, female sex, obesity, diabetes, and renal disease,[36, 37] there are currently no class I guideline recommended treatments for HFpEF that improve mortality.[38, 39] HFpEF has received increased attention since HFpEF patients frequently experience delayed diagnosis and have limited treatment options. Recent studies have shown that HFpEF is a clinically heterogeneous disorder, consisting of subgroups with related comorbidities and pathophysiologies, which lead to different progression trajectories.[40, 41] Employing latent class analytics and clustering techniques based on widely available clinical variables, several investigators have shown that patients with HFpEF can be divided into distinct classes with differing outcomes.[40, 41] "

 "CVD progression can be affected by both gene and environment via different molecular pathways and mechanisms. Although the high throughput technologies have enabled accurate and cost-effective genotyping in large population samples, comparable high throughput measurements of environmental exposures are needed for large population studies. Gene-environment interaction is a common mechanism to explain complex disease risk, and inter-individual variability. Better understanding of gene-environment interactions and their causal relationships will point to pathways and mechanisms as potential targets for treatment and intervention. The gene-environment interaction study may also help understand the CVD risk among immigrant populations exposure to different environmental and lifestyle factors."

# Peer review: Reviewer response for version 1

**DOI:** 10.21956/wellcomeopenres.17960.r41143

**Published:** 2020-11-13

**Authors:** Chunyu Liu

**Affiliations:** 1 Department of Biostatistics, Boston University, Boston, MA, USA

Cardiovascular disease (CVD) is the leading cause of mortality in South Asia, with rapidly increasing prevalence of hypertension, type 2 diabetes and hyperlipidemia over the last two decades. A thorough understanding of CVD-associated omics profiles may lead to more accurate estimates of CVD progression and stimulate new strategies for improving cardiovascular health in South Asia. However, I don’t think this review provided a comprehensive review for multi-omics study of CVD and risk factors. Below is the summary of my comments.

1. The title of this review is missing leading. When I read the title ‘Cardiovascular disease risk and pathophysiology in South Asians: can longitudinal multi-omics shed light’ I thought this review summaries omics research of CVD and risk factors in South Asia. But the paper mostly based on participants of European origin. Therefore, this is really a perspective of future research in Asia. The title is not appropriate.
2. I suggest the paper comprehensively summarized the research publications in the field. For example, for blood pressure GWAS, the paper cited 2011 GWAS. There are at least 4 GWAS publications after 2015. For DNA methylation, there are many papers with larger sample sizes published after 2015. But this paper only included a few for CVD risk factors.
3.  If talking about multi-omics, the paper should also include publications of CVD and risk factors with proteomics and gene expression/RNA seq.
4. I also suggest the co-authors include a table summarize the resent publications for CVD outcomes and risk factors.

Is the review written in accessible language?

Yes

Are all factual statements correct and adequately supported by citations?

Yes

Are the conclusions drawn appropriate in the context of the current research literature?

Yes

Is the topic of the review discussed comprehensively in the context of the current literature?

Partly

Reviewer Expertise:

Statistical genetics and genomics.

I confirm that I have read this submission and believe that I have an appropriate level of expertise to confirm that it is of an acceptable scientific standard, however I have significant reservations, as outlined above.

# Response

**Published:** 2021-05-07

**Authors:** Yan Sun

**Affiliations:** Emory University Rollins School of Public Health, USA

*1. The title of this review is missing leading. When I read the title ‘Cardiovascular disease risk and pathophysiology in South Asians: can longitudinal multi-omics shed light’ I thought this review summaries omics research of CVD and risk factors in South Asia. But the paper mostly based on participants of European origin. Therefore, this is really a perspective of future research in Asia. The title is not appropriate.*

**The reviewer is correct about the perspective component of the manuscript because the multi-omics research of CVD is still limited, particularly among South Asians. Meanwhile, the prevalence of heart failure and atherosclerotic heart disease in South Asian populations is rising, and the incidence is higher at younger age and leaner body mass index in South Asians compared to European population. The growing evidence from epidemiologic studies emphasizes the importance of improving cardiovascular health among South Asians. Therefore, we acknowledge that the current omics research of CVD is primarily conducted among European population, and address the needs for such omics studies in South Asians. We also noted a recent genetic study of coronary heart disease, which demonstrated the predictive ability of polygenic risk score in South Asians (PMID 32762905). As a result, we hope the title represents the current knowledge of CVD in South Asians, multi-omics of CVD (mostly in European ancestry) and anticipation for the future efforts in tackling the disparity in South Asians, a mixture of review and perspective in the present manuscript.**

*2. I suggest the paper comprehensively summarized the research publications in the field. For example, for blood pressure GWAS, the paper cited 2011 GWAS. There are at least 4 GWAS publications after 2015. For DNA methylation, there are many papers with larger sample sizes published after 2015. But this paper only included a few for CVD risk factors*.

**We appreciate the reviewer’s suggestion on including recent publications of large GWAS and DNA methylation studies. We included recent blood pressure GWAS with larger sample sizes (PMID 30224653, PMID 30578418, PMID 30429575), in addition to the 2011 blood pressure GWAS as an early multi-ethnic GWAS including South Asians (PMID 21909115). We also added recent multi-ethnic GWAS publications of CVD and risk factors, including coronary heart disease (PMID 33532862, PMID 33020668, 2021 preprint DOI: 10.21203/rs.3.rs-275591/v1), heart failure (PMID 31919418, PMID 30586722), type 2 diabetes (PMID 32541925), BMI (PMID 30124842), and blood lipids (PMID 30275531). The revised text under “Genetic basis of atherosclerotic cardiovascular disease and heart failure” is listed below:**

 "Genome-wide association studies (GWAS) have identified a large number of genetic loci associated with coronary heart disease,[42-44] ASCVD,[45-47] HF,[48, 49] and their risk factors, such as BMI,[50, 51] blood lipids,[52, 53] blood pressure,[54-57] and T2DM;[58] however, these genetic variations explain only a small portion of risk in populations.[59, 60]"

**When reviewing the epigenome-wide association studies (EWAS), we focused on studies with prospective design and potential causal inference. For example, the Agha 2019 study (PMID 31424985) discovered CpG sites associated with incident coronary heart disease or myocardial infarction, and the Mendelian Randomization analysis revealed the causal effect of DNA methylation sites in the disease mechanism. The Westerman’s 2019 paper (PMID 31615550) also discovered DNA methylation modules associated with incident cardiovascular disease. Additionally, we included several large EWAS of CVD risk factors, including type 2 diabetes (PMID 31506343, PMID 26095709), BMI (PMID 28002404), blood lipids (PMID 28194238) and blood pressure (PMID 31999706, PMID 32520614, PMID 29198723). The revised text under “Epigenetic signatures identify molecular pathways of CVD that are activated in the context of environmental risk” is listed below:**

 "EWAS provides an unbiased approach for identifying molecular mediators of genetic and environmental factors that may explain residual risk of disease[74] and has been applied to investigate CVD[75-79] and risk factors.[80-85]"

 "The study of incident T2DM conducted among Indian Asians and Europeans showed a 2.5 times higher adjusted risk among Indian Asians than Europeans, and five loci including ABCG1, PHOSPHO1, SOCS3, SREBF1, and TXNIP were associated with incident T2DM among Indian Asians and replicated among Europeans.[81] The other study of incident T2DM reported additional loci such as PHGDH and CPT1A, which were discovered among Europeans and replicated among Indian Asians.[80] The study of blood pressure compared the methylation profiles between Europeans and South Asians revealed many distinct loci between the two ancestries with a small overlap.[84] To our current knowledge, some known trans-ethic epigenetic loci might be transportable from Europeans to South Asians, but there is still a lack in studies specifically among large South Asian populations to further explore novel loci to explain the higher risk. A number of EWAS have examined DNAm patterns associated with air pollution, which is a known environmental risk factor for CVD.[97,98]"

**For the multi-omics studies, we included a recent publication that integrated genome-wide single-nucleotide polymorphisms, gene expression, and DNA methylation, revealed the molecular mechanism of heart failure (PMID 31703168), and another study of CVD (PMID 33836805). The revised text under “The multi-omics approach is critical to understand CVD risk at the molecular level” is listed below:**

 "A recent study using longitudinal big data including genome, immunome, transcriptome, proteome, metabolome, microbiome and wearable monitoring have showed the potential of revealing cardiovascular pathophysiology on a molecular basis.[118] Although increasing number of population studies have measured multi-omics data, recent studies have focused on single -omic association study and used additional -omics data to better understand the molecular functions of identified associations.[119] Integrated multi-omics studies of CVD outcomes have been limited. A large HF study of over 8,000 participants within the Framingham Heart Study utilized integrative trans-omics data including genetic variations, DNA methylation, and gene expression data to reveal genetic contributions towards HF. The transportability of such findings to South Asians needs to be evaluated.[120] Another study integrating DNA methylation and gene expression data identified independent latent factors associated with CVD. The unsupervised machine learning of multi-omics successfully improved classification and discrimination.[121]"

*3. If talking about multi-omics, the paper should also include publications of CVD and risk factors with proteomics and gene expression/RNA seq.*

**We appreciate the suggestion. We have added a paragraph summarizing “Proteomics and gene expression reveal the interactions between cell processes and external environment”. The text under this section is listed below:**

 "The proteomics technologies have evolved rapidly in the past two decades. Recent applications of proteomics in population studies have produced interesting findings in CVD research.[99] A recent study of the healthy human heart tissue collected from autopsy determined the healthy heart proteome. The resulted database, which included over 10,700 proteins, is a comprehensive resource for the downstream investigations.[100] In a targeted proteomics study, two protein signatures for high-risk plaques and absence of coronary atherosclerosis were identified among a cohort with suspected coronary artery disease.[101] The prediction accuracy of a model constructed by 50 proteins showed a better prediction accuracy in adverse cardiovascular events than a traditional risk factor model.[102] These findings are critical in CVD risk prediction and differentiation of CVD subtypes. In addition, gene expression profiling in CVD also enables a better understanding of pathophysiology of CVD.[103] Patterns of gene expression of human aorta tissue was investigated to identify genes with prediction power in atherosclerosis.[104] Studies of non-coding transcriptome, which even though have limited protein-coding functions, are discovering the pathology of CVD. To eventually realize precision medicine, we are still facing the challenges of standardization of methodologies and translation.[105] In addition, researches in this area largely depend on the availability of tissues from autopsy, thus the clinical translation and implementation has been limited. However, such studies facilitate a better understanding on the disease causal pathway and mechanism. Particularly, there has been a lack of similar researches particularly among South Asians, which is urging the future efforts of investigation."

*4. I also suggest the co-authors include a table summarize the resent publications for CVD outcomes and risk factors.*

**We appreciate the suggestion. As we focused on the CVD outcomes in South Asians, we added a summary table (Table 1) for recent omics studies of CVD including coronary artery disease, stroke, myocardial infarction and heart failure, particular in multi-ethnic studies when available. We also added text in the “Introduction” section.**
